# Prediction of Allograft Failure in Pediatric Kidney Transplant Recipients: A Validation Study of the Four-Variable Kidney Failure Risk Equation

**DOI:** 10.7759/cureus.72901

**Published:** 2024-11-02

**Authors:** Sara Nogueira Machado, Joana Freitas, Célia Sofia Moreira, Ana Teixeira, Teresa Costa, Maria Sameiro Faria, Maria Conceição Mota

**Affiliations:** 1 Pediatrics, Unidade Local de Saúde do Alto Ave, Guimarães, PRT; 2 Nephrology, Unidade Local de Saúde de Santo António, Porto, PRT; 3 Mathematics, Faculdade de Ciências da Universidade do Porto, Porto, PRT; 4 Pediatric Nephrology, Centro Materno-Infantil do Norte, Unidade Local de Saúde de Santo António, Porto, PRT

**Keywords:** children, graft failure, prediction, risk equation, transplant

## Abstract

Introduction: Recent findings suggest that the four-variable Kidney Failure Risk Equation (KFRE) may be useful in predicting the likelihood of allograft failure in adult kidney transplant (KT) recipients. However, research on its application in pediatric patients is lacking. This study aimed to assess the accuracy of the four-variable KFRE for prediction of the two-year and five-year risk of allograft failure in a cohort of pediatric KT recipients.

Methods: A retrospective observational study of patients undergoing KT in a tertiary pediatric nephrology unit between 2007 and 2017 was conducted. The KFRE risk scores were determined using data collected one-year post-transplantation. Discrimination and calibration properties of the four-variable KFRE were assessed through the area under the receiver operating characteristic curves (AUC) and calibration plots.

Results: Fifty-nine patients with a median age of 12.4 (9.1-15.7) years at KT were included. Eleven (18.6%) were living donor recipients. The median estimated glomerular filtration rate one-year post-transplantation was 62.0 (49.0-75.0) mL/min/1.73 m^2^. One (1.7%) and three (5.1%) patients experienced allograft failure within two and five years following the one-year post-transplantation date, respectively. The four-variable KFRE showed excellent and very good discrimination for the two-year and five-year risks, respectively (AUC 0.966, 95% confidence interval (CI) 0.914-1.000; AUC 0.887, 95% CI 0.732-1.000). Calibration plots demonstrated imprecise calibration.

Conclusion: The four-variable KFRE shows promise in predicting kidney failure progression in pediatric KT recipients with a functioning allograft one-year post-transplantation. Larger-scale studies are essential to confirm its predictive accuracy and establish more definitive conclusions.

## Introduction

Kidney transplantation is the lead treatment for end-stage renal disease (ESRD) in children, as it offers significantly better long-term outcomes than dialysis [1]. Nevertheless, allograft failure is still a pressing issue when it comes to transplant complications, despite all medical progress relating to improving its management [2]. In patients with a failing transplant, it’s primal to stratify risk in order to guarantee that both patients and their families are adequately prepared for the potential need for kidney replacement therapy [3].

The Kidney Failure Risk Equation (KFRE) was established in 2011 by Tangri et al. [4] to assist clinical professionals in their decision-making when it comes to adults with chronic kidney disease (CKD). The KFRE is a four-variable model that predicts the two-year and five-year likelihood of progression to ESRD requiring kidney replacement treatment. This simple and easily calculated risk score relies on age, sex, estimated glomerular filtration rate (eGFR), and urine albumin-to-creatinine ratio (uACR) [4]. Since its development and validation in multiple cohorts of adults [5], the KFRE has shown high discriminative performance in estimating the two-year and five-year risk of ESRD in a heterogeneous group of children with CKD [6] and was thus recommended for pediatric practice [7].

Recently, the KFRE has also been shown to accurately predict allograft failure in adult kidney transplant (KT) recipients at one-year post-transplantation [8,9]. However, to present, the diagnostic accuracy of the KFRE in pediatric patients who underwent a KT has not yet been assessed. If proven effective in predicting allograft failure in those same patients, the KFRE could be a valuable tool in monitoring, following up, and timely preparing for kidney replacement therapy in those at higher risk of allograft failure [8]. Herein, the present study aimed to assess the accuracy of the four-variable KFRE in predicting the two-year and five-year risk of allograft failure in a cohort of pediatric KT recipients with a functioning allograft at one-year post-transplantation.

This study was previously presented as a focused oral communication at the 61st ERA Congress on May 24, 2024. In accordance with the Congress rules, the abstract was published in the journal Nephrology Dialysis Transplantation (Volume 39, Issue Supplement 1, https://doi.org/10.1093/ndt/gfae069.982).

## Materials and methods

Study design and sample

A single-center retrospective observational cohort study was performed. All patients under 18 years of age who underwent a KT in a tertiary pediatric nephrology unit in northern Portugal between January 1, 2007, and December 31, 2017, who had at least one uACR measurement and one serum creatinine measurement approximately one-year post-transplantation, were included. Patients who died or reached allograft failure (defined as eGFR <15 mL/min/1.73 m^2^, starting hemodialysis or peritoneal dialysis, or receiving another KT) within the first year post-transplantation were excluded.

Data collection and variables

Clinical data was obtained from the individual electronic records of the patients included in the study.

The risk of kidney failure at two and five years following the one-year post-transplantation date was estimated for each patient using the four-variable KFRE. The eGFR was calculated using the creatinine-based revised bedside Schwartz equation [10]. The uACR was estimated from the urine protein-to-creatinine ratio (uPCR) for some patients, using a publicly available online conversion tool [11]. All variables needed to calculate the KFRE risk scores were obtained as close to the one-year post-transplantation date as possible (and limited up to 18 months post-transplantation).

Death-censored allograft failure, established as eGFR <15 mL/min/1.73 m^2^, starting hemodialysis or peritoneal dialysis, or undergoing retransplantation, was the primary outcome. The KFRE-predicted kidney failure risks were compared to the actual outcomes at two and five years following the one-year post-transplantation date.

Statistical analysis

Continuous variables are presented as medians and interquartile ranges. For categorical variables, data is presented in the form of frequency and percentage. For the comparison of baseline characteristics between living and deceased donor recipients, logistic and robust analyses were performed, respectively, for dichotomous and continuous variables.

To evaluate the performance of the four-variable KFRE in predicting allograft failure using patient data collected one-year post-transplantation, its discrimination and calibration properties were analyzed. Discrimination refers to a model’s ability to effectively distinguish high-risk from low-risk patients. Receiver-operator characteristic curves were generated, and discrimination was then defined as the area under the curve (AUC) with 95% confidence intervals (CI). The optimal threshold was calculated using Youden’s J statistic. An AUC of 1.0 signifies perfect discrimination, while an AUC of 0.5 suggests the model performs no better than random chance [12]. Calibration, on the other hand, assesses the difference between predicted risk and actual outcomes. Perfect calibration, whereby the predicted probabilities match the observed events, is represented by a 45º line on a calibration plot [12].

Statistical analyses were conducted using RStudio, R version 4.3.2, and the packages: pROC, rms, tidymodels, and robustbase (RStudio Team. (2015). RStudio: Integrated Development Environment for R. Boston, MA. Retrieved from http://www.rstudio.com/). A p-value of less than 0.05 was accepted as statistically significant.

Ethical considerations

This study was approved by the Comissão de Ética Santo António, Instituto de Ciências Biomédicas Abel Salazar (approval number: 2024-082).

## Results

Patient characteristics

A total of 59 pediatric patients were included in the study. Demographic characteristics and clinical data at one-year post-transplantation are represented in Table 1.

**Table 1:** Demographic characteristics and clinical data of the study cohort at one-year post-transplantation Continuous data are presented as median (interquartile range) and categorical data as frequencies (percentages). CKD: chronic kidney disease, eGFR: estimated glomerular filtration rate, NR: not reported, uACR: urine albumin-to-creatinine ratio, uPCR: urine protein-to-creatinine ratio ^^ ^following the one-year post-transplantation date, ^* ^p<0.1

Variable	All patients (n=59)	Living donor recipients (n=11)	Deceased donor recipients (n=48)	p-value
Age				
At the time of the transplant (years)	12.4 (9.1-15.7)	13.2 (9.1-16.0)	12.0 (8.5-15.4)	0.307
At the time analytical data was collected (years)	13.6 (10.1-16.8)	15.5 (10.1-17.6)	13.1 (9.5-16.7)	0.309
Male sex	41 (69.5)	10 (90.9)	31 (64.6)	0.119
Cause of CKD				
Nonglomerular	46 (78.0)	10 (90.9)	36 (75.0)	0.274
Glomerular	13 (22.0)	1 (9.1)	12 (25.0)	
Laboratory findings				
eGFR (mL/min/1.73 m^2^)	62.0 (49.0-75.0)	57.0 (46.0-63.0)	64.5 (50.0-80.8)	0.051^*^
uPCR (mg/g)	235.5 (127.9-401.4)	235.5 (97.8-245.9)	235.6 (151.4-452.8)	0.175
uACR (mg/g)	57.0 (23.0-137.0)	60.0 (15.0-94.5)	54.5 (27.5-147.6)	0.277
Outcome				
Graft failure within 2 years^^ ^	1 (1.7)	0 (0,0)	1 (2.1)	0.998
Graft failure within 5 years^^^	3 (5.1)	2 (18.2)	1 (2.1)	0.066^*^
Time to graft failure (years)	10.0 (4.0-13.0)	4.1 (3.9-NR)	11.5 (6.4-13.3)	0.334

Most patients were male (n=41, 69.5%), with a median age of 12.4 (9.1-15.7) years at the time of the KT. The majority (n=46, 78.0%) had a nonglomerular cause of CKD. The analytical measurements were taken at a median of 1.2 (1.1-1.3) years after the transplant. Median eGFR at one-year post-transplantation was 62.0 (49.0-75.0) mL/min/1.73 m^2^. Of the 59 patients, 11 (18.6%) were living donor recipients, who exhibited a tendency for lower eGFR at one-year post-transplantation (p=0.051).

Throughout the follow-up period, one (1.7%) and three (5.1%) patients reached the primary outcome of allograft failure within two and five years following the one-year post-transplantation date, respectively. In this cohort, living donor recipients had approximately ten times higher odds of experiencing allograft failure five years after the one-year post-transplantation date compared to deceased donor recipients (p=0.066).

KFRE performance: discrimination

The four-variable KFRE showed excellent discriminative ability for the two-year risk (AUC 0.966, 95% CI 0.914-1.000) and very good discrimination for the five-year risk (AUC 0.887, 95% CI 0.732-1.000) of allograft failure, as detailed in Table 2. The optimal cut-off points for the KFRE were identified as 5.8% and 2.3% for the two-year and five-year risks of allograft failure, respectively. Patients surpassing these thresholds are expected to achieve allograft failure.

**Table 2:** Discrimination statistics of KFRE AUC: area under the receiver-operator characteristic curve, CI: confidence interval, KFRE: Kidney Failure Risk Equation ^ following the one-year post-transplantation date

Performance measure	2-year risk^^^	5-year risk^^^
AUC (95% CI)	0.966 (0.914-1.000)	0.887 (0.732-1.000)
Sensitivity	100.0%	100.0%
Specificity	96.6%	73.2%
Threshold (%)	5.81	2.27

KFRE performance: calibration

**Figure 1:**
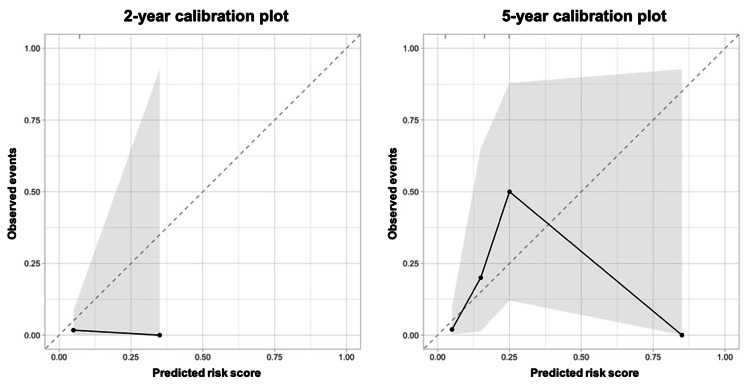
Calibration plots

The calibration plots, as shown in Figure 1, revealed imprecise calibration for the four-variable KFRE in the transplant cohort at two and five years following the one-year post-transplantation date.

## Discussion

While significant progress has been made in exploring predictive models of allograft failure in pediatric patients with CKD [13,14], a robust and effective model is yet to be established. This study aimed to actively contribute to this ongoing pursuit by assessing the four-variable KFRE’s accuracy in predicting allograft failure among pediatric KT recipients surviving one-year post-transplantation.

While the present study demonstrates that the four-variable KFRE exhibits adequate discriminative ability in predicting the two-year and five-year risks of allograft failure, it also reveals its imprecision with respect to calibration. This implies that, while effectively distinguishing between high- and low-risk patients, there are notable incongruities between predicted and observed risks. Notably, recent studies in various cohorts of adult KT recipients have reported a similar trend, with the four-variable KFRE displaying miscalibration despite its satisfactory discriminatory capacity [3,15]. Unfortunately, this miscalibration may significantly limit the reliability and applicability of KFRE in clinical settings, as calibration performance is often deemed the most important characteristic of a risk prediction tool [12].

It is noteworthy to mention that this study also explored an additional facet that enhances the clinical utility of the four-variable KFRE. The identification of optimal cut-off points provides tangible thresholds beyond which patients may be at a heightened risk of graft failure. By pinpointing these critical markers, this research contributes not only to the understanding of predictive accuracy but also to the practical applicability of the four-variable KFRE in a clinical context.

In contrast to prevailing literature [2], the presented results suggest a greater likelihood of allograft failure in living donor recipients; however, caution should be applied to the interpretation of this finding due to the limited number of observed graft failures in this sample.

To the authors’ knowledge, this study marks the first assessment of the four-variable KFRE’s predictive performance in pediatric KT recipients. Despite yielding promising results, it is crucial to acknowledge its limitations. First and foremost, it is critical to note that the KFRE was not originally designed for transplant patients, and as such it does not account for alloimmune factors, such as donor type and characteristics, human leukocyte antigen antibodies, histopathology characteristics, and immunological parameters, all of which are known to influence the progression to allograft failure [16]. Additionally, the model was initially developed and validated in patients with eGFR <60 mL/min/1.73 m2, a threshold slightly lower than the median value of the present cohort [4]. Secondly, the small sample size and low event rate likely influenced the KFRE calibration, limiting the generalizability of the presented results. Thirdly, while the authors successfully estimated some uACR using a validated conversion tool with high correlation [11], this approach may have affected the predicted risk scores. Lastly, the study’s retrospective nature may have also impacted the accuracy of clinical data collection. Future research should prioritize larger, multi-center studies to validate and refine the KFRE model, addressing the identified limitations and ensuring a more representative sample.

## Conclusions

The four-variable KFRE demonstrates the potential for predicting kidney failure in pediatric KT recipients with a functioning allograft at one-year post-transplantation. However, large-scale studies are needed to validate these findings and confirm the strength of the herein-presented conclusions. Further research should also explore the applicability of this model across different demographic and clinical subgroups to ensure its generalizability and efficacy in diverse settings.
